# Individual Differences in the Neural Basis of Response Inhibition After Sleep Deprivation Are Mediated by Chronotype

**DOI:** 10.3389/fneur.2019.00514

**Published:** 2019-05-15

**Authors:** Jingjing Song, Pan Feng, Xin Wu, Bingbing Li, Yanchen Su, Yingjiang Liu, Yong Zheng

**Affiliations:** Key Laboratory of Cognition and Personality (MOE), Faculty of Psychology, Southwest University, Chongqing, China

**Keywords:** chronotype, sleep deprivation, response inhibition, interindividual difference, go/no-go task, inferior frontal gyrus, functional magnetic resonance imaging

## Abstract

Sleep deprivation (SD) has been reported to severely affect executive function, and interindividual differences in these effects may contribute to the SD-associated cognition impairment. However, it is unclear how individual differences in chronotypes (morning-type, MT; evening-type, ET) influence neurobehavioral functions after SD. To address this question, we used functional magnetic resonance imaging (fMRI) to evaluate whether 24 h of SD differentially affect response inhibition, a core component of executive function, in MT and ET individuals. Accordingly, MT and ET participants were instructed to follow their preferred 7–9-h sleep schedule for 2 weeks at home both prior to and throughout the course of the study, and then performed a go/no-go task during fMRI scanning at 08:00 a.m. both at rested wakefulness (RW) and following SD. We also examined whether the neurobehavioral inhibition differences in the chronotypes in each session can be predicted by subjective ratings (sleepiness, mood, and task) or objective attention. Behaviorally, SD led to an increased response time of go trials (hit RT), more attentional lapses, higher subjective sleepiness, and worse mood indices, but it did not impair the accuracy of go trials (hit rate) and no-go trials (stop rate). Regardless of the presence of SD, ET individuals exhibited a lower stop rate, higher subjective ratings of sleepiness, exhausted mood, and task difficulty in comparison with MT individuals. On the neural level, SD resulted in decreased inhibition-related activation of the right lateral inferior frontal gyrus (rIFG) in MT individuals and increased rIFG activation in ET individuals. Moreover, the rIFG activation in ET individuals after SD was positively correlated to the subjective ratings of sleepiness and effort put into the task, which was considered as a compensatory response to the adverse effects of SD. These findings suggest that individual differences in inhibition-related cerebral activation after SD are influenced by chronotypes. In addition, ET individuals may be vulnerable to response inhibition. Thus, it is essential to take into consideration the chronotype in SD research and sleep medicine.

## Introduction

Sleep deprivation (SD) is commonplace in modern society, and there is increasing neuroimaging evidence suggesting that the prefrontal cortex may be particularly susceptible to the impacts of sleep loss due to its extensive use during normal waking (1). Accordingly, SD should particularly impair complex executive functions that rely on the prefrontal regions (2). However, studies on this assumption have yielded inconsistent results, with some groups reporting impairments in executive function tasks during SD (3–5) and others failing to find such effects (6, 7). These inconsistencies may be attributable to interindividual differences. For instance, earlier studies reported that individuals who are better able to maintain inhibitory efficiency exhibit a larger activation in the prefrontal cortex as a compensatory response to SD relative to those whose inhibitory efficiency declines (8).

The concept of chronotype relies on the subjective preference for activities in the morning or evening (morning- [MT] or evening- [ET] type). MT individuals are most alert in the early morning and prefer to go to sleep and wake up early. By contrast, ET individuals are most alert toward later in the evening and prefer to go to sleep and wake up late (9, 10). Individuals with different chronotypes differ in their homeostatic sleep regulation; the build-up (11) and dissipation (12) rate of sleep pressure are faster in MT individuals than in ET individuals. Even under conditions of sleep fragmentation (5-min awakenings every 30 min), MT individuals exhibit increased homeostatic response (13). In a normal waking day, ET individuals are more capable of maintaining alertness (14) and executive function (15) by recruiting arousal-promoting brain structures with increasing homeostatic sleep pressure. However, few studies have directly examined the interindividual chronotype differences (MT vs. ET) in the neurobehavioral responses to an elevation in sleep pressure triggered by total SD (16). Therefore, the present study employed functional magnetic resonance imaging (fMRI) to evaluate whether 24-h total SD differentially affects the neurobehavioral differences in response inhibition, a core component of executive function, between MT and ET individuals. To investigate this question, MT and ET participants underwent scanning while performing a go/no-go task in both rested wakefulness (RW) and SD conditions. Furthermore, the study aimed to examine whether the subjective ratings (sleepiness, mood, and task) and objective attention (psychomotor vigilance) of chronotypes in each session reflect the neurobehavioral differences in response inhibition.

On the basis of previous findings, we expected that SD impairs inhibition-related neurobehavioral responses, such as poor inhibition performance (8) and impaired frontoparietal network activities (17), which are especially located in the right lateral inferior frontal gyrus (rIFG) and critical for successful response inhibition (18, 19). Furthermore, we hypothesized that SD would differentially impact the neurobehavioral changes of chronotypes and that the subjective ratings and objective attention of participants in each session would predict to some extent the neurobehavioral responses to inhibition.

## Methods

### Subjects

Participants were recruited from students who completed the self-reported Morningness–Eveningness Questionnaire (20) at Southwest University. The inclusion criteria were as follows: (1) age, 18–30 years; (2) normal or corrected-to-normal vision; (3) right-handedness; and (4) a regular sleep-wake schedule that includes 7–9 h of total sleep time. Exclusion criteria were as follows: (1) self-reported history of psychiatric, neurologic, or sleep disorders; (2) drug or alcohol abuse, excessive caffeine (>5 cups of coffee per day) or nicotine (>5 cigarettes per day) intake; (3) travel across more than two time zones within 3 months before the study; and (4) presence of contraindications for fMRI.

The chronotype was determined by the Chinese version of the Morningness–Eveningness Questionnaire (21, 22) which has good psychometric properties. After the answers had been checked and scored by the experimenters (score >62, MT participant; score < 50, ET participant), 26 MT individuals (mean score = 64.2 ± 3.4) and 22 ET individuals (mean score = 40.0 ± 4.2) were recruited for this study. Three participants (MT, 2; ET, 1) were excluded from data analysis because of excessive head movement and the presence of behavioral outliers. This study received approval from the Institutional Review Board at the Southwest University, Chongqing and followed the principles of the Declaration of Helsinki. All participants gave written informed consent before the experiment and were compensated for their participation.

### Experimental Procedure

The participants visited the laboratory three times. At their first visit, the participants underwent the screening process, were informed of the study requirements, and practiced the task. The participants filled out the Pittsburgh Sleep Quality Index [PSQI; (23)], Epworth Sleepiness Scale (24), the positive and negative affective schedule (25), the self-rating depression scale (26), the self-rating anxiety scale (27), the NEO Five-Factor Inventory (28), the Barrett Impulsiveness Scale-11 [BIS; (29)], and the Dysexecutive Questionnaire [DEX; (30)]. Only participants with regular sleep habits (self-reported sleep for 7–9 h per night) were invited to take part in the following experiments. The participants were then instructed to follow their preferred 7–9-h sleep-wake schedule at home for at least 2 weeks both prior to and throughout the course of the study. Compliance was verified by using sleep diaries. In addition, alcohol, nicotine, and caffeine intake, napping, and intense physical activity were forbidden for at least 24 h before scanning.

Participants were scanned twice with a week between the scans. The order of the two scanning sessions was randomized and counterbalanced (31, 32). The two sessions were conducted 1 week apart to minimize the possible residual effects of SD. In the RW session, participants underwent scanning at 08:00 a.m. after a night of normal sleep at home. Before the experiment, the participants were instructed to sleep about 7–9 h the night, get up at least 1 h prior to the beginning of scanning, and arrived at the laboratory at 07:30 a.m. to prepare for the following scanning. In the SD session, participants were monitored by the two experimenters in the laboratory from 10:00 p.m. until scanning began at 08:00 a.m. For both sessions, lighting conditions were carefully controlled at a steady low level, and exposure to sunlight was avoided. During the SD session, at every hour from 10:00 p.m., the participants performed the 10-min version of the Psychomotor Vigilance Task [PVT; (33, 34)], responded to the Karolinska Sleepiness Scale [KSS; (35)] as well as a mood-related Likert-type rating scale (range, 0–10) which was defined by the items motivated–unmotivated, fresh–exhausted, elated–depressed, congenial–irritable, relaxed–stressed, and calm–anxious (8). For the rest of the time, participants were kept awake with non-strenuous activities like reading, watching movies, and conversing with the experimenters. In addition, regular snacks were available. Prior to the scanning, subjects carried out the PVT, KSS, the mood rating, and had a task practice. Then, participants performed the task immediately in the fMRI scanner. After task completion but still in the scanner, participants were asked to complete the KSS, the mood rating, and the 10-point Likert scales (36) to assess the following task-related factors: task difficulty, ability to concentrate, effort put into the task, and motivation to perform the task well. The number of lapses (RTs >500 ms) and mean RT in PVT were treated as indexes of psychomotor vigilance (33).

### Task

**Figure 1:**
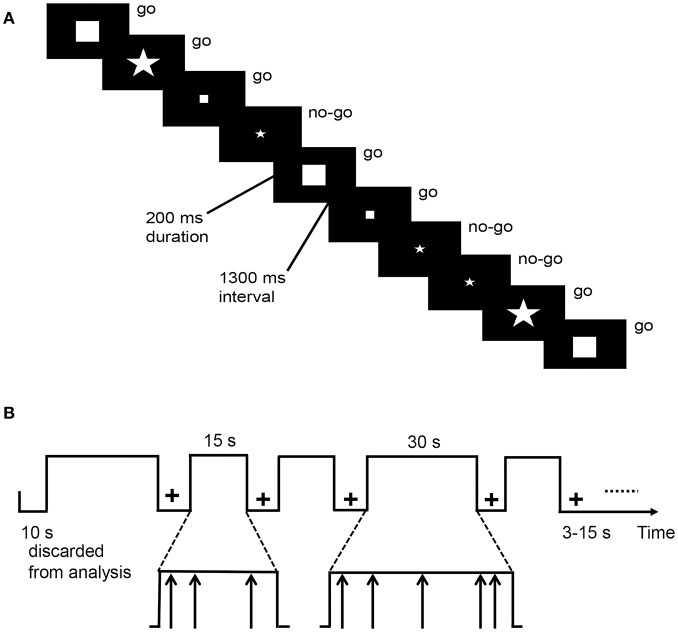
Schematic representation **(A)** and the timing parameters **(B)** of the go/no-go task.

The go/no-go task requires from the participant continual responses to stimuli while bearing in mind to refrain from responding to a specific but less frequently presented stimulus. This task [Figure 1; (36, 37)] alternated between task blocks and resting blocks. During resting blocks, a fixation cross appeared in the center of the screen. During task blocks, stimuli were exhibited by an event-related design and four shapes (go stimuli: large square, small square, large pentagon; the no-go stimulus: small pentagon) were presented one at a time in the center of the screen. Once the subjects observed a go stimulus, they had to press a button using the right finger as soon as possible. However, they were required to refrain from responding when they observed the no-go stimulus. Task blocks were in total 270 s long, in which each of five task blocks lasted 30 s and another eight blocks lasted 15 s. Resting blocks were in total 114 s and varied between 3 and 15 s (mean = 8.8 s). Stimuli appeared for 200 ms every 1,500 ms. There were 180 stimuli in total, of which 75% were go stimuli. The task lasted 6 min 24 s. The response time of go trials (hit RT), as well as the accuracy of go (hit rate) and no-go (stop rate) trials, were assessed.

### fMRI Data Acquisition

Images were collected on a 3-Tesla MR scanner (Siemens Magnetom Trio TIM; Erlangen, Germany). A magnetization-prepared gradient echo sequence was employed to acquire T1-weighted anatomical images: TR = 1,900 ms, TE = 2.52 ms, flip angle = 9°, FOV = 250 × 250 mm^2^, in-plane resolution = 0.98 × 0.98 mm, slices = 176, thickness = 1 mm. A single-shot gradient echo-planar imaging (EPI) sequence was employed to acquire task-based functional T2^*^-weighted images: TR = 2,000 ms, TE = 30 ms, bandwidth = 2,232 Hz/pixel, flip angle = 90°, FOV = 220 × 220 mm^2^, matrix size = 64 × 64, slices = 32, thickness = 3 mm, inter-slice gap = 1 mm.

### fMRI Data Analysis

We used SPM12 and DPABI2.1 to analyze the functional data (38, 39). For each participant, the first five images were discarded due to non-steady magnetization, the rest of the functional images were corrected for slice timing and spatially realigned using six parameters of head motion. The structural images were co-registered to the EPI mean image and segmented into white matter, gray matter, and cerebrospinal fluid. The functional data were normalized to a Montreal Neurological Institute (MNI) space with a voxel size of 3 × 3 × 3 mm^3^ and spatially smoothed using a Gaussian kernel with 8 mm full width at half maximum.

In the first-level analysis, a statistical analytical design was estimated using the general linear model (GLM). Five regressors were created (rest; go success, GS; go error, GE; no-go success, NGS; and no-go error, NGE) after convolution with the canonical hemodynamic response function [HRF; (40)]. Six realignment parameters were included in the model to attribute to the residual variance, and a high-pass filter of 128 s was used to remove possible effects of low-frequency changes. Sex and age differences were controlled as covariates. Subsequently, we probed the inhibition-related cerebral activations using the contrast of NGS and GS at the group level [two-tailed Gaussian random field correction, voxel level: *p* < 0.001, cluster level: *p* < 0.05; (41)]. The group analysis targeted the interaction effects between chronotype and session on response inhibition. In addition, using functional MRI results, the regions-of-interest (ROIs) were defined by a sphere of 6 mm radius around the centers of the peak coordinates of inhibition-related areas. For the ROI analysis using MarsBar (42), individual β values were extracted. To assess the interaction effect on response inhibition, repeated-measures analysis of variance (ANOVA) based on the ROIs were carried out using SPSS Statistics 20.0, followed by Tukey's *post hoc* tests.

## Results

### Participants

The features of the participants are specified in Table 1. Global and several component (sleep quality, latency, and disturbance; Table S1) scores on the PSQI were significantly higher for ET individuals, with higher scores indicating more severe complaints. In addition, ET individuals showed marginally significantly higher values for the non-planning factor [*t*_(43)_ = −1.81, *p* = 0.08] on the BIS and the inhibition factor [*t*_(43)_ = −1.78, *p* = 0.08] on the DEX (Table S1). According to the sleep diaries (Table 2), chronotypes showed significant differences in sleeping and waking time but not in sleep duration.

**Table 1:** Participant characteristics.

**Measures**	**MT (*N* = 24)**	**ET (*N* = 21)**	***p*-value**
Sex(male/female)	8/16	8/13	
Age	21.29 ± 2.37	20.14 ± 1.24	
PSQI	3.75 ± 1.42	5.90 ± 2.44	< 0.001
ESS	11.38 ± 3.99	11.38 ± 3.34	0.99
**PANAS**			
Positive affect	30.79 ± 5.70	30.24 ± 7.08	0.77
Negative affect	18.54 ± 6.01	20.38 ± 6.65	0.34
SDS	44.17 ± 8.53	47.38 ± 9.73	0.24
SAS	33.96 ± 5.21	36.43 ± 7.01	0.18

*PSQI, Pittsburgh Sleep Quality Inventory; ESS, Epworth Sleepiness Scale; PANAS, positive and negative affective schedule; SDS, self-rating depression scale; SAS, self-rating anxiety scale*.

**Table 2:** Sleep characteristics based on sleep diary.

**Measures**	**MT (*N* = 24)**	**ET (*N* = 21)**	***p*-value**
Sleep onset time of 1st week	23:44 ± 0:30	0:54 ± 0:35	< 0.001
Wake-up time of 1st week	7:03 ± 0:41	8:52 ± 2:08	< 0.001
Sleep duration of 1st week^+^	7.30 ± 0.76	7.41 ± 0.73	0.630
Sleep onset time of 2nd week	23:39 ± 0:32	0:53 ± 0:40	< 0.001
Wake-up time of 2nd week	7:22 ± 1:00	8:24 ± 0:50	< 0.001
Sleep duration of 2nd week^+^	7.60 ± 0.730	7.56 ± 0.78	0.857

*MT, morning-type; ET, evening-type; M, 24-h clock time; SD, “hours: minutes”; ^+^, sleep duration is in hours*.

### Behavioral Findings-Subjective and Objective Measures

For the go/no-go task, repeated-measures ANOVAs for the accuracy and RT data were carried out (Table 3). A main effect of session was noted on the hit RT [*F*_(1, 43)_ = 4.34, *p* < 0.05] after controlling for the covariates of sex and age, i.e., the hit RT was significantly higher following SD than at RW. However, the hit rate [*F*_(1, 43)_ = 2.43, *p* = 0.13] and stop rate [*F*_(1, 43)_ = 0.27, *p* = 0.61] showed no significant main effects of session. A main effect of chronotype was found on the stop rate [*F*_(1, 43)_ = 8.65, *p* < 0.01] after controlling for the covariates of sex and age. In other words, ET individuals showed a significantly lower stop rate than MT individuals. However, the hit rate [*F*_(1, 43)_ = 0.18, *p* = 0.68] and hit RT [*F*_(1, 43)_ = 0.01, *p* = 0.93] showed no significant main effects of chronotype. The interaction effect of chronotype and session on hit rate [*F*_(1, 43)_ = 0.34, *p* = 0.57], hit RT [*F*_(1, 43)_ = 0.52, *p* = 0.48], and stop rate [*F*_(1, 43)_ = 0.25, *p* = 0.62] were not significant.

**Table 3:** Accuracy and reaction times of the go/no-go task according to session and chronotype.

	**Morning-type**		**Evening-type**	
	**RW**	**SD**	**RW**	**SD**
Hit rate (%)	0.97 ± 0.07	0.92 ± 0.14	0.97 ± 0.06	0.87 ± 0.15
Hit RT (ms)	434.52 ± 64.59	443.38 ± 69.30	420.32 ± 86.83	456.39 ± 80.65
Stop rate (%)	0.91 ± 0.07	0.88 ± 0.11	0.82 ± 0.13	0.77 ± 0.13

*RW, rested wakefulness; SD, sleep deprivation; Hit rate, the accuracy of go trials; Hit RT, the reaction times of go trials; Stop rate, the accuracy of no-go trials*.

**Figure 2:**
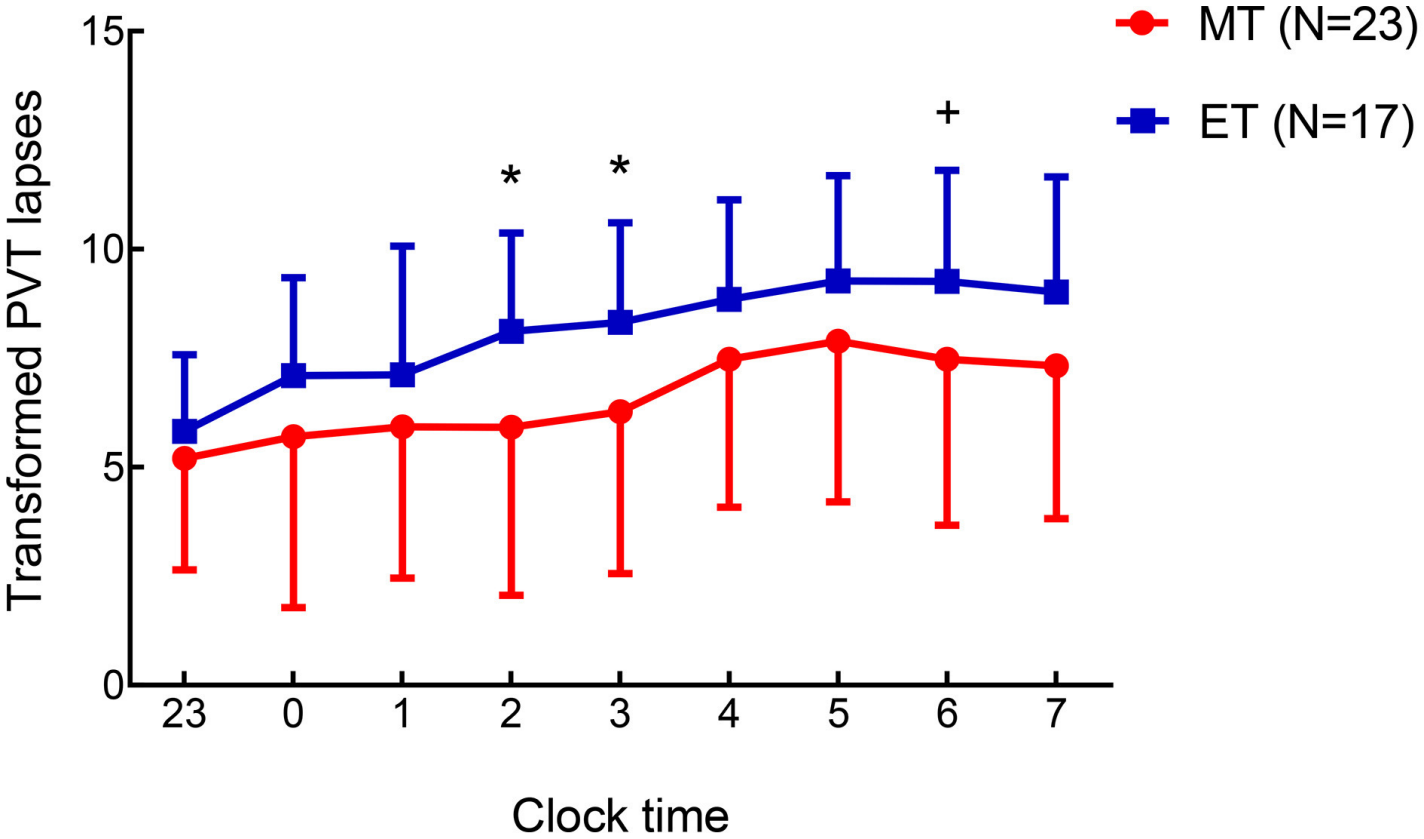
Mean ± standard deviation change in the transformed Psychomotor Vigilance Task (PVT) lapses ($\sqrt{\text{lapses}}+\sqrt{\text{lapses}+1}$) determined hourly during the period from 11:00 p.m. to 07:00 a.m. on the 24-h sleep deprivation (SD) night in morning-type (MT) and evening-type (ET) participants. The differences (MT vs. ET) on transformed PVT lapses were investigated using *t*-tests for independent samples. Five participants were eliminated from the analysis (1 from the MT, 4 from the ET) owing to failure to complete the PVT hourly at the SD night. Condition effect *p < 0.05; +: the effect was marginally significant.

With respect to the PVT, we focused on the number of lapses [transformed lapses: $\sqrt{\text{lapses}}\;+\sqrt{\text{lapses}+1}$; (33)] as the primary outcome to assess vigilance. Repeated-measures ANOVAs (Table S2) showed a significant main effect of session [*F*_(1, 43)_ = 67.66, *p* < 0.001] and a marginally significant main effect of chronotype [*F*_(1, 43)_ = 3.88, *p* = 0.06] on the transformed lapses. As expected, the number of lapses after SD were more than those after RW. In addition, more lapses (trend level) occurred in ET individuals than in MT individuals. Next, to explore the differences in lapses during SD between MT and ET individuals, an independent-samples *t*-test (MT vs. ET) was performed on transformed lapses (values between 11:00 p.m. and 07:00 a.m.) during the SD session (Figure 2). Five subjects were eliminated from the analysis (1 from the MT and 4 from the ET) since they failed to complete the PVT hourly on the SD night because of a technical error. The findings revealed more lapses among ET individuals at 02:00 a.m. [*t*_(1, 38)_ = −2.10, *p* < 0.05], 03:00 a.m. [*t*_(1, 38)_ = −2.17, *p* < 0.05], and 06:00 a.m. [*t*_(1, 38)_ = −1.78, *p* = 0.08, marginally significant] compared to MT individuals (Figure 2). Thus, ET individuals may be at a disadvantage while completing the PVT in the SD condition.

A repeated-measures ANOVA was also performed on the subjective measures (ratings of sleepiness, mood, and task). The ratings for sleepiness and each item on mood just before each scanning session differed significantly between the two sessions (Table S2). In comparison with RW, subjects presented increased sleepiness and decreased mood parameters after SD. We also observed a main effect of session on ratings of sleepiness, mood, and task [effect on task difficulty was marginally significant, *F*_(1, 43)_ = 3.38, *p* = 0.07] during the scanning (Table S3). Thus, SD significantly changed almost all subjective ratings of sleepiness, mood, and task. Importantly, the main effect of chronotype on the ratings of sleepiness [*F*_(1, 43)_ = 10.38, *p* < 0.01], fresh–exhausted mood [*F*_(1, 43)_ = 4.91, *p* < 0.05], and task difficulty [*F*_(1, 43)_ = 9.52, *p* < 0.01] were statistically significant, with ET individuals showing significantly higher values than MT individuals.

### fMRI Results

A repeated-measures ANOVA for chronotype (MT vs. ET) and session (RW vs. SD) revealed a significant interaction effect between the two factors (two-tailed Gaussian random field correction, voxel level: *p* < 0.001, cluster level: *p* < 0.05) in the right lateral inferior frontal gyrus region [rIFG: Brodmann area 46, peak coordinate (45, 54, 12); peak intensity: 18.26; number of voxels: 47; Figure 3A], which was closely associated with the execution of response inhibition.

**Figure 3:**
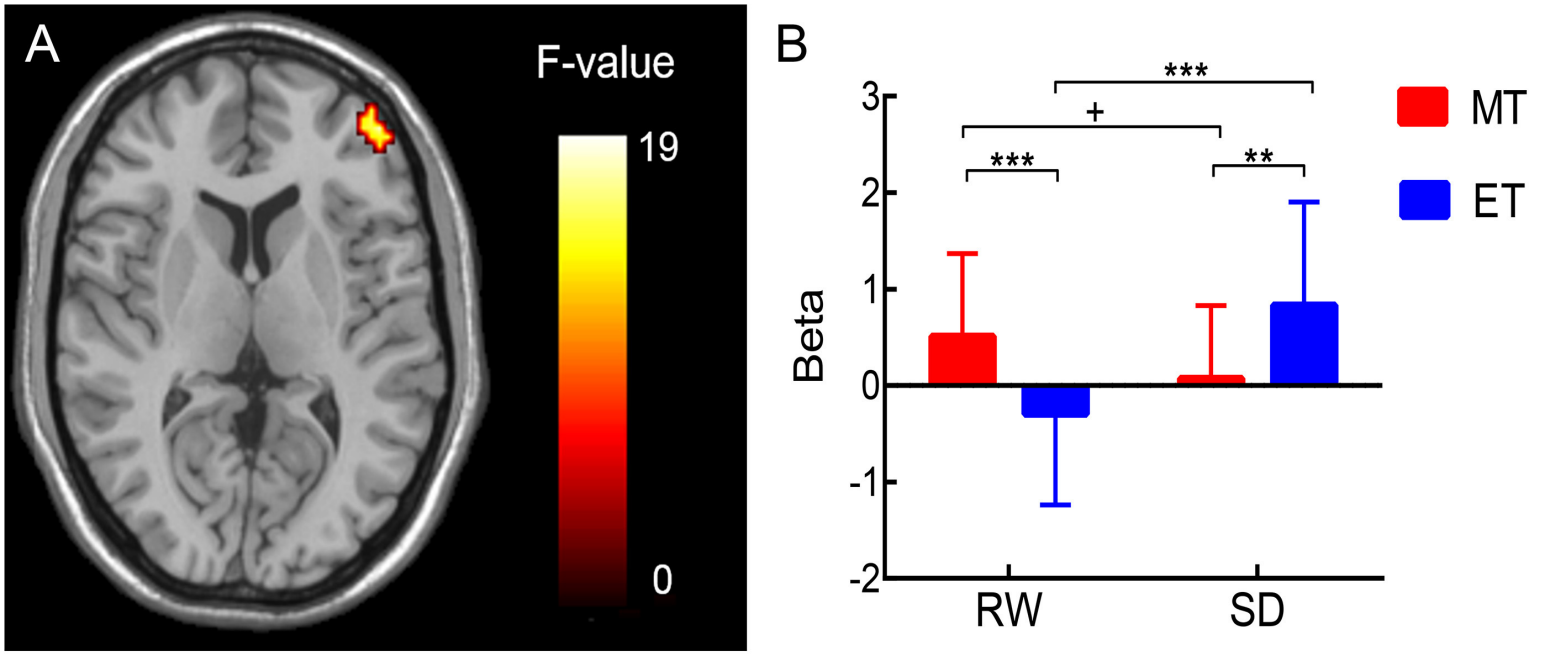
**(A)** Brain regions showing an interaction effect between chronotype and session during response inhibition (NGS vs. GS) in the right lateral inferior frontal gyrus [rIFG; Brodmann area 46, peak coordinate (45, 54, 12); peak intensity: 18.26; number of voxels: 47; two-tailed Gaussian random field correction, voxel level: *p* < 0.001, cluster level: *p* < 0.05]. **(B)** For ROI analysis, the results showed that inhibition-related (NGS vs. GS) response in rIFG decreased from the rested wakefulness (RW) session to the sleep deprivation (SD) session in morning-type (MT) participants, whereas rIFG activity significantly increased from the RW session to the SD session in evening-type (ET) participants. Condition effect **p < 0.01, ***p < 0.005; +: the effect was marginally significant.

For the ROI analysis, there was a significant chronotype × session interaction effect on the rIFG region [*F*_(1, 43)_ = 17.86, *p* < 0.001; Figure 3B]. Then, a simple effect analysis were performed. The results (Figure 3B) were as follows: in MT participants, the cerebral responses induced by successful inhibition (NGS > GS) in the rIFG decreased [*t*_(1, 23)_ = 2.01, *p* = 0.06] in SD compared to RW sessions; in ET participants, the rIFG activity significantly increased [*t*_(1, 20)_ = −3.66, *p* < 0.005] in SD compared to RW sessions. In addition, rIFG activity in MT participants was significantly higher compared with ET participants at RW [*t*_(1, 43)_ = 3.02, *p* < 0.005], whereas rIFG activity in ET participants was significantly higher compared with MT participants after SD [*t*_(1, 43)_ = −2.78, *p* < 0.01].

### Brain-Behavior Correlation Results

**Figure 4:**
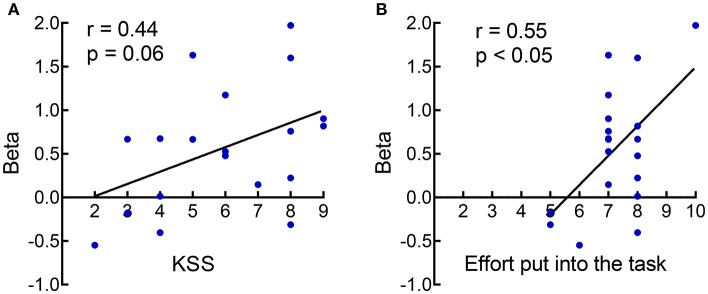
The correlation analysis showed that the rIFG activity in ET participants was positively related to subjective ratings of sleepiness [**(A)**; Karolinska Sleepiness Scale, KSS] and the effort put into the task **(B)** after SD.

To examine whether the subjective ratings (sleepiness, mood, and task-related factors) and objective task performance (PVT and go/no-go task) are predictive of the inhibition-related rIFG activation in each session, we performed a correlation analysis between behavioral indices (subjective ratings, objective task performance) and rIFG activation at RW and following SD in the two chronotypes. In MT individuals, rIFG activity was positively correlated with subjective ratings for task difficulty (*r* = 0.45, *p* < 0.05) and negatively correlated with hit RT (*r* = −0.43, *p* < 0.05) during the RW session. However, rIFG activity and behavior indices showed no correlation during the SD session. Among ET individuals, rIFG activity was negatively correlated to the mean RT of the PVT during the RW session (*r* = −0.45, *p* < 0.05) and positively correlated to subjective ratings of sleepiness just before the scanning (*r* = 0.44, *p* = 0.06) and the effort put into the task (*r* = 0.55, *p* < 0.05; the correlation analysis was computed with 19 data pairs due to two outliers of neural activity in the rIFG) during the SD session (Figure 4). Obviously, a relationship between rIFG activity and attention (hit RT of the go/no-go task or mean RT of the PVT) was only detectable during RW but not after SD. This suggests that SD altered the association between inhibition-related activation and attention.

## Discussion

We investigated the interindividual differences in the neurobehavioral functions associated with response inhibition between MT and ET individuals after SD. Behaviorally, SD led to an increase in hit RT, more attentional lapses, increased sleepiness, and worse mood indices. However, SD did neither impair the hit rate nor the stop rate. Regardless of the presence of SD, ET individuals demonstrated a lower stop rate, as well as higher subjective ratings of sleepiness, exhausted mood, and task difficulty compared to MT individuals. On the neural level, SD resulted in decreased inhibition-related rIFG activation in MT individuals and in increased rIFG activation in ET individuals. Moreover, the rIFG activation in ET individuals after SD was positively correlated to the subjective ratings of sleepiness and effort put into the task. These findings suggest that ET individuals demonstrate an increased rIFG activation after SD, which is consistent with previous studies (8, 37) and can be interpreted as a compensatory response to SD. Together, the present findings thus provide a new chronotype-related perspective to explore the differential SD-induced effects on cognition.

Consistent with the results of previous studies (37, 43), the subjects in this study experienced a significant increase in hit RT after SD. Studies have shown that SD leads to a general slowing of response times of attention (44, 45), and a meta-analysis reported that after SD, reaction time is more vulnerable than accuracy (46). As expected, the increase in hit RT was accompanied by increased lapses in vigilance, greater sleepiness, and worse mood, which is consistent with previous research (43, 47). SD did not impair the inhibition performance in the study, which could be attributable to the fact that scanning was not performed in the early morning hours which are regarded as a sensitive time-window for SD (48). Studies have suggested that SD should particularly impair executive functions (2, 3), such as response inhibition (5, 8, 37, 43). By contrast, other studies have shown that the inhibition performance does not differ between RW and SD sessions (49, 50). In the future, more studies are necessary to identify which components of executive functions are reliably impaired by sleep loss.

Interestingly, in comparison to MT individuals, ET individuals showed a significantly lower stop rate during RW and following SD. This finding suggested that ET individuals were probably particularly vulnerable to response inhibition. Furthermore, ET individuals scored higher in non-planning factors on the BIS and the inhibition factor on the DEX. Previous studies have shown that ET individuals are correlated with increased impulsivity, enhanced disinhibition, and impaired response inhibition (trend level) in comparison to MT individuals (51–53). However, another study could not confirm an effect of chronotype on the inhibition performance assessed by the stop-signal task in a synchronous effect paradigm (54). We hypothesized that differences in task paradigm, experimental design, and study population may have contributed to the dissimilarity in findings. Additionally, the heterogeneous factors defining impulsivity, which represents a broad concept, predict psychological outcomes (55), and inhibitory control is not a unitary construct (56). Considering the complex interactions between behavioral inhibitory control and the self-reported trait impulsivity (56), future studies should further examine the detailed relationships among chronotype, inhibitory control, and impulsivity (57).

In our study, ET individuals also scored higher than MT individuals in the subjective ratings for sleepiness, exhausted mood, and task difficulty, regardless of whether the participants underwent SD. Additionally, ET individuals exhibited in comparison to MT individuals a worse subjective sleep quality as assessed by the PSQI, which is consistent with previous findings (58–60). We hypothesized that the increases in sleepiness and exhausted mood in ET individuals, which may be attributable to poorer sleep quality, could have contributed to the higher ratings for task difficulty and the impaired inhibition performance. Due to common social standards in everyday life, ET individuals have to work in the morning which conflicts with their preferred time of activities. This social jetlag, i.e., the asynchrony between social and biological rhythms, occurs chronically throughout an individual's learning and working life (61), which directly leads to less sleep in ET individuals on weekdays (62–64) and probably influences the sleep quality and pattern. Therefore, environmental factors such as early work or school starting times may result for ET individuals in sleep and circadian disturbances like social jetlag or disturbed sleep that act on neuropsychological mechanisms such as response inhibition or impulsivity (65). Moreover, researchers highlight the importance of utilizing longitudinal studies to specifically determine the direction of effects among chronotypes, social jetlag, and psychological outcomes in the future (66).

On the neural level, the whole-brain activation results indicated significant interaction effects in the rIFG region which is consistent with prior studies (36, 37). Previous findings suggest that the role of the rIFG is critical for inhibiting response trends (18) and is related to both response and attentional control (67, 68). The rIFG has also been characterized as a “brake,” and it can be initiated in both total (to outright suppress a response) and partial (to pause the response) conditions (18).

Crucially, the current study revealed significant interaction effects of chronotype and SD on the cerebral activation patterns of response inhibition, i.e., a decreased rIFG activation pattern in MT participants but an increased rIFG activation in ET participants for SD in comparison to RW. The prevailing hypothesis proposes that functioning of the frontal lobe is particularly affected by sleep loss (1). In the present study, a decreased prefrontal activity during SD was only apparent in MT participants. By contrast, ET participants demonstrated increased rIFG activation following SD, which was positively related to subjective ratings of sleepiness (trend level) and effort put into the task. Researchers have insisted that effort is closely linked to the concept of motivation, and the degree of effort is probably particularly elevated when individuals are motivated or perceive the trends of poor task performance (69). In addition, the PVT findings indicated more attentional lapses among ET individuals during the SD night, especially at 02:00 a.m., 03:00 a.m., and 06:00 a.m. (trend level), compared to the corresponding values for MT individuals. Thus, the effects of SD appear to differ across cognitive domains in the two chronotypes. ET individuals may show vulnerabilities on sustained attention, but could also show increased inhibition-related cerebral activation as a compensatory response after SD. However, the results were inconsistent with the previous study (16), in which participants were instructed to follow a fixed sleep-wake schedule (different from personal preferred sleep schedules). Then, both chronotypes performed a simple reaction time test hourly during 36 h of extended wakefulness under constant routine. Consequently, ET individuals maintained optimal alertness (fastest 10% reaction time) throughout the night, but MT individuals did not. We surmised that differences in experimental design, study population, and dependent variable may have contributed to the dissimilarity in findings. Importantly, the individual differences of chronotypes in sustained attention after SD should be explored in a larger sample size in the future. Finally, we hypothesized that the ET individuals in our study probably experienced a negative impact on attention after SD. In addition, these individuals demonstrated increased sleepiness, more exhausted mood, higher task difficulty, and poorer inhibition performance, thus needing to put more effort into the task which leads to increased rIFG activation acting as a compensatory response. In SD session, the time of testing started at 08:00 a.m. which differed from the preferential time of ET individuals. This adverse circadian time may have exaggerated the compensatory trend. However, this response in ET individuals was not sufficient to reverse the adverse effects on inhibition performance in SD. Alternatively, the short task duration caused differences in cerebral activation after SD without detectable behavioral changes (48). By contrast, MT individuals exhibited relatively decreased sleepiness, fresh mood, lower task difficulty, and better inhibition performance. We hypothesized that MT individuals may have experienced only a subtle negative SD impact and detected that the task was not difficult, hence it was unnecessary to increase the brain activation to maintain performance.

Recent studies have persistently indicated significant individual differences in homeostatic sleep responses and cognitive performances after SD (70, 71), and the *PERIOD3* (*PER3*) polymorphism has been related to individual differences after total SD (72, 73). Compared to individuals with the shorter allele (*PER3*^4/4^), those expressing the longer allele (*PER3*^5/5^) showed a greater cognitive decline (72–74) and a more widespread reduction in task-related (working memory task, 3-back) cortical activations following SD (75). It has been suggested that *PER3*^5/5^ may mediate the differential susceptibility via its impact on sleep homeostasis (72, 76). Furthermore, the *PER3* polymorphism is correlated with the chronotype, *PER3*^5/5^ is associated with MT and *PER3*^4/4^ with ET (77–79), although the correlation of chronotypes and *PER3* genotypes is not consistently verified (80, 81). Meanwhile, it is essential to note an assumption in which the interactions among genotype, phenotype, and social constraints should be taken into consideration (48). In the present study, ET individuals perhaps exhibited vulnerabilities on sustained attention after SD, which was probably influenced by social constraints such as social jetlag. Compared with the abundance of studies focusing on individual genotype-related differences after SD, studies addressing phenotype-related differences are relatively rare. To better reflect real-life situations and to acquire more information about social jetlag, it is essential to pay more attention in the future to individual chronotype-linked differences in SD-related neurobehavioral studies, which could be assessed using the Munich Chronotype Questionnaire (82).

The present results should be understood in the context of several limitations. First, the sample size in our study was relatively small. Therefore, the results need to be verified in a larger sample. Second, the task were scheduled according to external clock time, not according to the personalized preferential waketime of the participants. Consequently, the masking effects on circadian and homeostatic processes for the two chronotypes were not fully controlled. Moreover, the physiological circadian and homeostatic indicators of participants were not assessed in the study. Therefore, an exact correlation of the SD-induced inhibition impairment with the chronotype-associated circadian and homeostatic changes could not be established. Future studies should schedule the testing periods according to individual time plans and consider utilizing a combination of techniques from the fields of physiology, psychology, and cognitive neuroscience, especially when focusing on a chronotype-related difference in the SD paradigm. Third, the total SD is probably not the most appropriate paradigm for chronotypes, and future studies should verify the differences in chronotypes by means of a chronic sleep restriction paradigm. It will also be interesting to explore the chronotype-related differences in recovery from sleep loss. Many studies have targeted the differences between subjects in susceptibility to the neurobehavioral changes after SD within a cognitive domain, and future studies should pay more attention to within-subjects and between-domain differences in susceptibility (83).

In summary, these findings indicate that ET individuals were vulnerable to inhibition, and the poorer inhibition performance was accompanied by higher subjective ratings of sleepiness, exhausted mood, and task difficulty. In addition, ET individuals exhibited a worse attention performance on the SD night. Importantly, the individual differences in inhibition-related cerebral activation after SD were influenced by chronotypes, with decreased rIFG activation in MT individuals, but increased rIFG activation in ET individuals, which was considered as a compensatory mechanism to cope with the SD-induced adverse effects such as more attentional lapses, although changes in regional responses preceded the behavioral modifications in the present study. Accordingly, it is essential to take into consideration the chronotype in SD-related neurobehavioral research and sleep medicine in the future.

## Ethics Statement

This study was approved by the Institutional Review Board at the Southwest University, Chongqing and conformed to the tenets of the Declaration of Helsinki. All subjects provided written informed consent prior to the experiment and were compensated for their time.

## Author Contributions

JS, PF, XW, and YZ conceived and designed the study. JS, XW, BL, YS, and YL carried out the study. JS and PF performed the data analysis. JS wrote the main manuscript text whereas YZ, PF, and BL revised it critically prior to submission for publication.

### Conflict of Interest Statement

The authors declare that the research was conducted in the absence of any commercial or financial relationships that could be construed as a potential conflict of interest.
