# Supplementary material for: Individual Differences in the Neural Basis of Response Inhibition After Sleep Deprivation Are Mediated by Chronotype
Source: Front Neurol. 2019 May 15;10:514. doi: 10.3389/fneur.2019.00514 (PMC6529982; doi:10.3389/fneur.2019.00514)
Supplement: Supplementary file 1 [file Table_1.DOCX]

Table S1. Participant characteristics

| Measures | MT (N = 24) | ET (N = 21) | *p* value |
| --- | --- | --- | --- |
| heitht | 1.65±0.09 | 1.65±0.09 | 0.91 |
| weight | 55.75±7.63 | 59.33±13.84 | 0.28 |
| BMI | 20.47±2.10 | 21.51±2.72 | 0.16 |
| PSQI |  |  |  |
| Sleep quality | 0.88±0.68 | 1.48±0.60 | <0.005 |
| Latency | 0.92±0.72 | 1.57±0.87 | <0.01 |
| Duration | 0.29±0.55 | 0.33±0.48 | 0.79 |
| Sleep efficiency | 0.125±0.34 | 0.05±0.22 | 0.37 |
| Disturbance | 0.79±0.41 | 1.19±0.40 | <0.005 |
| Use of medications | 0.00±0.00 | 0.24±0.77 | 0.17 |
| Daytime dysfunction | 0.75±0.61 | 1.05±0.74 | 0.15 |
| NEO |  |  |  |
| Neuroticism | 30.96±7.39 | 33.48±7.97 | 0.28 |
| Extraversion | 37.79±6.97 | 38.43±6.45 | 0.75 |
| Openness to experience | 43.46±5.40 | 41.95±5.95 | 0.38 |
| Agreeableness | 44.38±3.78 | 40.00±3.85 | <0.001 |
| Conscientiousness | 45.00±6.83 | 41.52±5.49 | **0.07** |
| BIS |  |  |  |
| Non-planning | 31.56±13.29 | 39.40±15.75 | **0.08** |
| Motor | 32.81±13.28 | 37.5±12.40 | 0.23 |
| Attention | 30.63±11.89 | 32.86±12.85 | 0.55 |
| Total score | 31.67±10.54 | 36.59±10.61 | 0.13 |
| DEX |  |  |  |
| Inhibition | 7.08±3.23 | 9.00±4.01 | **0.08** |
| intentionality | 6.54±3.09 | 7.14±2.54 | 0.48 |
| Knowing-doing dissociation | 5.46±2.67 | 5.90±2.51 | 0.57 |
| In-resistance | 5.54±2.62 | 6.57±2.93 | 0.22 |
| Social relulation | 2.13±1.23 | 2.67±1.20 | 0.14 |

MT= morning-type; ET= evening-type; BMI = Body Mass Index; PSQI = Pittsburgh Sleep Quality Inventory; NEO = NEO Five-Factor Inventory; BIS = Barrett Impulsiveness Scale; DEX = Dysexecutive Questionnaire.

In bold: the effect was marginally significant.

Table S2. The means and standard deviations (Std) of the objective PVT performance, subjective ratings of sleepiness, and mood performed just before each scanning are reported as a function of chronotype (MT vs. ET) and session (RW vs. SD). The F values represent the effect of chronotype, session, and their interaction on these measures, which were investigated using repeated-measures ANOVA.

|  | Mean ± Std | | F value | | |
| --- | --- | --- | --- | --- | --- |
| Measures | MT | ET | chronotype | session | Chronotype × session |
| PVT |  |  |  |  |  |
| Lapses-RW^a^ | 4.17±2.00 | 5.21±2.62 | **3.875** | 67.657*** | 0.816 |
| Lapses-SD^a^ | 6.77±3.16 | 8.46±2.49 |  |  |  |
| Mean RT-RW^b^ | 2.54±0.04 | 2.56±0.04 | 1.491 | 46.457*** | 0.045 |
| Mean RT-SD^b^ | 2.58±0.04 | 2.59±0.04 |  |  |  |
| KSS-RW | 2.83±1.17 | 2.86±2.31 | 0.186 | 52.866*** | 0.187 |
| KSS-SD | 5.71±2.60 | 6.10±2.19 |  |  |  |
| Mood |  |  |  |  |  |
| Motivated-RW | 1.50±1.10 | 2.43±2.25 | 1.527 | 36.770*** | 0.398 |
| Motivated-SD | 4.38±2.58 | 4.76±2.90 |  |  |  |
| Fresh-RW | 1.62±1.21 | 2.43±1.89 | 2.639 | 53.676*** | 0.006 |
| Fresh-SD | 4.75±2.72 | 5.62±2.82 |  |  |  |
| Elated-RW | 2.79±1.44 | 3.10±1.81 | 0.971 | 54.690*** | 0.406 |
| Elated-SD | 4.87±1.96 | 5.57±2.62 |  |  |  |
| Congenial-RW | 2.29±1.37 | 2.86±1.82 | 0.711 | 26.087*** | 0.185 |
| Congenial-SD | 4.21±2.15 | 4.48±2.60 |  |  |  |
| Relaxed-RW | 2.29±1.60 | 2.52±1.81 | 0.046 | 22.836*** | 0.135 |
| Relaxed-SD | 4.13±2.03 | 4.10±2.41 |  |  |  |
| Calm-preRW | 1.83±1.52 | 2.10±1.51 | 0.016 | 29.835*** | 0.389 |
| Calm-preSD | 3.75±2.56 | 3.62±2.22 |  |  |  |

MT = morning-type, ET = evening-type; RW = rested wakefulness, SD = sleep deprivation; KSS = Karolinska Sleepiness Scale; Motivated = motivated–unmotivated, Fresh = fresh–exhausted, Elated = elated–depressed, Congenial = congenial–irritable, Relaxed = relaxed–stressed, Calm = calm–anxious.

^a^ ANOVA conducted on transformed lapses ($\sqrt{\mathrm{lapses}}$+$\sqrt{lapses+1}$) of PVT.

^b^ ANOVA conducted on Mean RT (log transformed) of PVT.

In bold: the effect was marginally significant.

Condition effect *** *p* < .001.

Table S3. The means and standard deviations (Std) of the ratings of sleepiness, mood, and task-related measures during the scanning session are reported as a function of chronotype (MT vs. ET) and session (RW vs. SD). The F values represent the effect of chronotype, session, and their interaction on these measures, which were investigated using repeated-measures ANOVA.

|  | Mean ± Std | | F value | | |
| --- | --- | --- | --- | --- | --- |
| Measures | MT | ET | chronotype | session | Chronotype × session |
| KSS-RW | 3.29±1.00 | 4.24±1.77 | 10.383** | 60.621*** | 1.309 |
| KSS-SD | 5.42±2.23 | 7.10±1.70 |  |  |  |
| Mood |  |  |  |  |  |
| Motivated-RW | 2.08±1.44 | 2.71±1.52 | 1.234 | 30.231*** | 0.082 |
| Motivated-SD | 4.25±2.40 | 4.67±2.48 |  |  |  |
| Fresh-RW | 2.13±1.30 | 2.95±1.60 | 4.911* | 62.118*** | 0.360 |
| Fresh-SD | 4.29±1.97 | 5.48±2.29 |  |  |  |
| Elated-RW | 2.63±1.41 | 3.00±1.58 | 0.744 | 46.308*** | 0.000 |
| Elated-SD | 4.67±2.04 | 5.05±2.01 |  |  |  |
| Congenial-RW | 2.54±1.38 | 2.81±1.29 | 0.021 | 54.789*** | 0.523 |
| Congenial-SD | 4.92±2.18 | 4.76±1.41 |  |  |  |
| Relaxed-RW | 2.42±1.32 | 2.81±1.60 | 0.009 | 32.739*** | 1.006 |
| Relaxed-SD | 4.79±2.23 | 4.48±1.83 |  |  |  |
| Calm-RW | 2.04±1.46 | 2.57±1.69 | 0.626 | 34.595*** | 0.239 |
| Calm-SD | 4.29±2.42 | 4.48±1.94 |  |  |  |
| Task |  |  |  |  |  |
| Difficulty-RW | 2.29±1.08 | 4.05±2.01 | 9.524** | **3.378** | 1.394 |
| Difficulty-SD | 3.17±1.69 | 4.24±2.34 |  |  |  |
| Concentrate-RW | 8.00±1.82 | 7.71±1.23 | 1.726 | 13.219*** | 0.623 |
| Concentrate-SD | 6.96±2.39 | 6.10±1.95 |  |  |  |
| Effort-RW | 8.21±2.09 | 8.29±1.35 | 0.106 | 7.584** | 0.449 |
| Effort-SD | 7.54±2.23 | 7.19±0.98 |  |  |  |
| Motivation-RW | 8.46±1.77 | 8.24±1.73 | 0.505 | 5.529* | 0.097 |
| Motivation-SD | 7.88±2.13 | 7.48±1.12 |  |  |  |

MT = morning-type, ET = evening-type; RW = rested wakefulness, SD = sleep deprivation; KSS = Karolinska Sleepiness Scale; Motivated = motivated–unmotivated, Fresh = fresh–exhausted, Elated = elated–depressed, Congenial = congenial–irritable, Relaxed = relaxed–stressed, Calm = calm–anxious; Difficulty = task difficulty, Concentrate = ability to concentrate, Effort = effort put into the task, Motivation = motivation to perform the task well.

In bold: the effect was marginally significant.

Condition effect * *p* < .05; ** *p* < .01; *** *p* < .001.
